# HIV Prevalence and Antenatal Care Attendance among Pregnant Women in a Large Home-Based HIV Counseling and Testing Program in Western Kenya

**DOI:** 10.1371/journal.pone.0144618

**Published:** 2016-01-19

**Authors:** Samson Ndege, Sierra Washington, Alice Kaaria, Wendy Prudhomme-O’Meara, Edwin Were, Monica Nyambura, Alfred K. Keter, Juddy Wachira, Paula Braitstein

**Affiliations:** 1 Academic Model Providing Access to Healthcare (AMPATH) Program, Eldoret, Kenya; 2 Moi University, College of Health Sciences, School of Public Health, Eldoret, Kenya; 3 Indiana University, School of Medicine, Indianapolis, Indiana, United States of America; 4 Moi Teaching and Referral Hospital, Eldoret, Kenya; 5 Duke University, School of Medicine and Duke Global Health Institute, Durham, North Carolina, United States of America; 6 Moi University, College of Health Sciences, School of Medicine, Eldoret, Kenya; 7 University of Toronto, Dalla Lana School of Public Health, Toronto, Canada; 8 Indiana University, Fairbanks School of Public Health, Indianapolis, Indiana, United States of America; 9 Regenstrief Institute, Inc. Indianapolis, Indiana, United States of America; Queensland University of Technology, AUSTRALIA

## Abstract

**Objective:**

To describe the uptake of and factors associated with HIV prevalence among pregnant women in a large-scale home-based HIV counseling and testing (HBCT) program in western Kenya.

**Methods:**

In 2007, the Academic Model Providing Access to Healthcare Program (AMPATH) initiated HBCT to all individuals aged ≥13 years and high-risk children <13 years. Included in this analysis were females aged 13–50 years, from 6 catchment areas (11/08-01/12). We used descriptive statistics and logistic regression to describe factors associated with HIV prevalence.

**Results:**

There were 119,678 women eligible for analysis; median age 25 (interquartile range, IQR: 18–34) years. Of these, 7,396 (6.2%) were pregnant at the time of HBCT; 4,599 (62%) had ever previously tested for HIV and 2,995 (40.5%) had not yet attended ANC for their current pregnancy. Testing uptake among pregnant women was high (97%). HBCT newly identified 241 (3.3%) pregnant HIV-positive women and overall HIV prevalence among all pregnant women was 6.9%. HIV prevalence among those who had attended ANC in this pregnancy was 5.4% compared to 9.0% among those who had not. Pregnant women were more likely to newly test HIV-positive in HBCT if they had not attended ANC in the current pregnancy (AOR: 6.85, 95% CI: 4.49–10.44).

**Conclusions:**

Pregnant women who had never attended ANC were about 6 times more likely to newly test HIV-positive compared to those who had attended ANC, suggesting that the cascade of services for prevention of mother-to-child HIV transmission should optimally begin at the home and village level if elimination of perinatal HIV transmission is to be achieved.

## Introduction

A majority of pregnant women living with HIV in the world are from sub-Saharan Africa, and it is estimated that only 68% of them received antiretroviral therapy prophylaxis during pregnancy and delivery in 2013.[1] The challenges associated with effective prevention of mother-to-child transmission of HIV (PMTCT) and measuring its impact are numerous and multi-factorial, despite the notable HIV care advancements in this region.[1, 2] UNAIDS has targeted zero new HIV infections by 2030 including eliminating new HIV infections among infants born to HIV-positive mothers, and promoting the health status of mothers.[1, 3] To achieve these goals, the current recommendation by the World Health Organization (WHO), is to routinely test and treat all pregnant women with HIV as part of antenatal care.[4] The aim is to realize universal access to focused antenatal healthcare as well as promote healthy neonatal and maternal outcomes.

Typically, the PMTCT cascade begins from, and is integrated into, antenatal care (ANC) to ensure a high rate of case detection and optimal treatment coverage.[5–7] Through this integration, pregnant women attending ANC are routinely tested for HIV and those testing HIV-positive are supposed to be immediately provided with PMTCT interventions including HIV treatment. Unfortunately only 58% of women in Kenya are estimated to attend the minimum recommended four antenatal care visits, and 40% attend their first ANC visit after 6 months gestation.[8, 9] Evidence shows that HIV-positive women receiving combination antiretroviral prophylaxis during pregnancy, delivery and breastfeeding can reduce mother-to-child transmission to less than 5%.[10] In addition to PMTCT services which target pregnant women and offer a provider-initiated approach to HIV testing, there are a number of other strategies that provide pregnant women with opportunities to know their HIV status, including home-based counseling and testing (HBCT). [11, 12]

While testing uptake and HIV prevalence among pregnant women at the facility level is well documented through routine Ministry of Health and donor reporting requirements, as well as ANC-based HIV surveillance studies, [13] population-based HIV prevalence among pregnant women remains poorly described, particularly in sub-Saharan Africa. Home-based counseling and testing (HBCT) has been shown to be effective in promoting universal testing coverage in the general population and can enhance timely enrolment of HIV-infected persons into care.[11, 12, 14] The objective of this study was to describe population-based uptake of and factors associated with HIV testing and HIV prevalence among pregnant women in a large-scale home-based HIV counseling and testing (HBCT) program in western Kenya.

## Methods

### Study Area

The Academic Model Providing Access to Healthcare (AMPATH) program has enrolled >150,000 HIV-infected patients and currently provides HIV care and treatment to approximately 85,000 people in 143 Ministry of Health (MOH) facilities across western Kenya. In 2007, AMPATH initiated a home-based HIV counseling and testing (HBCT) program. A detailed description of the HBCT program is contained elsewhere.[15] Briefly, following community mobilization and sensitization activities, certified HIV counselors conduct home visits and offer HIV counseling and testing to all consenting persons 13 years and older as well as children less than 13 years of age whose mother is either dead, HIV-positive, or whose vital and/or HIV status is of unknown. Rapid HIV tests (Determine^™^ and Unigold ^™^) were performed on persons older than 18 months while children 18 months and younger were referred to AMPATH facilities for dryspot- DNA polymerase chain reaction (PCR) testing for HIV. Post-test counseling was offered to all persons tested and those found HIV-positive were referred for HIV care. Pregnancy status was documented for all women of reproductive age, ascertained by self-report. For those who were pregnant, their antenatal care attendance for the current pregnancy (yes/no) was documented.

### Study Population

This observational study utilized data collected during HBCT in Kapsaret, Burnt Forest, Webuye, Chulaimbo, Teso and Port Victoria catchment areas. All women aged 13–50 years were considered to be of reproductive age and were included in the analysis.

### Data Collection

Data were collected from November 15, 2008 to January 5, 2012 using electronic handheld data collection tools (Personal Data Assistants, Android Phones), into standardized forms. Data were synchronized to a central server and cleaned for quality by data assistants at the central facility in each catchment.

### Ethical Considerations

Ethical approval was obtained from the Institutional Research and Ethics Committee at Moi University School of Medicine and Moi Teaching and Referral Hospital (Kenya), Indiana University’s Institutional Review Board (United States), and University of Toronto’s Research Ethics Board (Canada). HBCT was rolled out as a clinical program and hence ethical approval was provided for retrospective analysis of de-identified data. Patient records were de-identified and anonymized prior to analysis.

### Analysis

Analyses were conducted using STATA SE version 12. The primary outcome variables considered were having previously tested for HIV (*yes* vs. *no*), previous HIV result (*positive* vs. *negative*), testing in HBCT (*accepted* vs. *refused*) and HBCT test result (*positive* vs. *negative*). Explanatory variables included: 1) socio-demographics factors: age per 5 year increase and marital status (*married/cohabiting* vs. other including *single/separated/ divorced/widowed*); 2) socio-economic factors included education level (*primary*, *secondary*, *tertiary* vs. *none*), employment status (*employed* vs. *unemployed*), income level (< = 5000 vs. >5000 Kenyan Shillings); 3) ANC attendance (*not yet attended ANC in this pregnancy* vs. *have attended ANC*); and HBCT catchment area (*Kapsaret*, *Webuye*, *Burnt Forest*, *Chulaimbo*, *Teso* and *Port Victoria*. We used a non-parametric test (Wilcoxon rank-sum test) to test the association between dichotomous outcomes and continuous independent variables, and Pearson’s Chi Square test for categorical variables. Logistic regression was performed to describe factors associated with the outcome variables (previously tested for HIV, previously known HIV-positive, testing uptake, and HIV prevalence). The Webuye catchment area was missing key covariates (education, employment, and income) as these data were not collected during HBCT in this catchment. Multivariate models using these variables were constructed after dropping data from Webuye. A covariate qualified to be included in the model if it caused at least 10% change in the effect of ANC on the outcome and/or was considered to be a potential confounder (i.e. age, marital status, education, income).

## Results

### Sociodemographics

Of the 245,180 females counseled and tested during HBCT, 119,981 (48.9%) were aged between 13–50 years and eligible for analysis. We dropped 303 (0.25%) women who had missing pregnancy status. As shown in Table 1, the overall median age of these women was 25 (*IQR*: 18–34) years, 61% were married or co-habiting, 27% had no education and another 54% had only completed primary school, 57% were employed, and 96% were receiving an income of less than 5000 Kenyan shillings per month (~$60 USD).

**Table 1:** Socio-demographic and socio-economic characteristic by pregnancy status.

Characteristics	All women N (%) 119,678 (100%)	Pregnant n (%) 7,396 (6.2%)	Not pregnant n (%) 112,282 (93.8%)	p-value
**Age in years (median, IQR)**	25 (18–34)	24 (21–29)	25 (18–35)	<0.001
**Marital status**				<0.001
Single	30,142 (29.5%)	815 (11.5%)	29,327 (30.8%)	
Cohabiting/Married	62,087 (60.7%)	6,014 (84.5%)	56,073 (58.9%)	
Separated/Divorced	4,587 (4.5%)	199 (2.8%)	4,388 (4.6%)	
Widowed	5,491 (5.4%)	89 (1.3%)	5,402 (5.7%)	
Missing	17,371 (14.5%)	279 (3.8%)	17092(15.2%)	
**Educational Level**				<0.001
None	19,430 (26.9%)	925 (21.4%)	18,505 (27.2%)	
Primary	39,363 (54.4%)	2,673 (61.7%)	36,690 (54.0%)	
Secondary	10,778 (14.9%)	580 (13.4%)	10,198 (15.0%)	
Tertiary	2,786 (3.9%)	154 (3.6%)	2,632 (3.9%)	
Missing	47,321(39.5%)	3,064(41.4%)	44,257(39.4%)	
**Employed (vs. not)**	40,751 (57.2%)	2,606 (60.9%)	38,145 (57.0%)	<0.001
Missing	48,487 (40.5%)	3116(42.1%)	45371(40.4%)	
**Income (Kenya Shillings) (< = 5000 vs. >5000)**	61,755 (96.4%)	3,762 (97.0%)	57,993 (96.3%)	0.017
Missing	55,557 (46.4%)	3518(47.6%)	52039(46.4%)	
**Catchment area**				<0.001
Burnt Forest	16,442 (13.7%)	991(13.4%)	15,451(13.8%)	
Chulaimbo	14,112 (11.8%)	747 (10.1%)	13,365 (11.9%)	
Webuye	46,707 (39.0%)	3,038 (41.1%)	43,669 (38.9%)	
Teso	11,470 (9.6%)	766 (10.4%)	10,704 (9.5%)	
Port Victoria	15,050 (12.6%)	1048 (14.2%)	14,002 (12.5%)	
Kapsaret	15,897 (13.3%)	806 (10.9%)	15,091(13.4%)	

There were 7,396 (6.2%) pregnant women in the population, of whom 2,995 (40.5%) had not yet attended ANC during their current pregnancy. Pregnant women were slightly younger in age compared to non-pregnant women, and were more likely to be married. Compared to non-pregnant women, a higher proportion of pregnant women had at least primary education and were slightly more likely to be employed (Table 2). The study catchment areas were primarily rural, with Webuye catchment representing the largest number of women in general. Women in Webuye were similar in age to other catchments but different in other respects: women in Webuye were more likely to be married (90% vs. 49%) and pregnant (6.5% vs. 6.0%), but less likely to have previously tested HIV-positive (5.9% vs. 11.8%).

**Table 2:** Testing history, testing uptake, and prevalence by pregnancy status.

Variables	All women N (%) 119,678 (100%)	Pregnant n (%) 7,396 (6.2%)	Not pregnant n (%) 112,282 (93.8%)	p-value
**Previously tested for HIV**	45,634 (38.1%)	4,599 (62.2%)	41,035 (36.6%)	<0.001
**Previously known HIV positive**	4,660 (10.2%)	269 (5.9%)	4,391 (10.7%)	<0.001
**Accepted HIV counseling in HBCT**	118,544 (99.1%)	7,353 (99.4%)	111,191 (99.1%)	<0.001
**Accepted HIV testing in HBCT**	115,101 (96.2%)	7,144 (96.6%)	107,957 (96.2%)	0.005
**Newly HIV positive in HBCT**	3,653 (3.1%)	241 (3.3%)	3,412 (3.1%)	0.281
**Overall HIV prevalence In HBCT**	8,313 (6.9%)	510 (6.9%)	7,803 (6.9%)	0.802

NB: The above analysis excluded Webuye catchment area

### HIV testing history

Only 38.1% of women had previously tested for HIV: 62.2% among pregnant women compared to 36.6% among those not-pregnant (p<0.001) (Table 3). Among those who had previously been tested, 10.2% of all women already knew they were HIV-positive (5.9% of pregnant women and 10.7% of non-pregnant women). In adjusted analysis, pregnant women were more likely to have previously tested for HIV if they were older, or had a primary or secondary school education. They were less likely to have previously tested if they were married or cohabiting, and had never attended ANC (Table 2).

**Table 3:** Factors associated with testing uptake among pregnant women.

Variables	Previously tested for HIV		Accepted testing in HBCT	
	UOR (CI)	AOR (CI)	UOR (CI)	AOR (CI)
**Age** per 5 year increase	**1.09 (1.03–1.15)**	**1.90 (1.03–1.17)**	**0.69 (0.63–0.75)**	**0.66 (0.60–0.72)**
**Marital Status** (married vs. not)	**0.65 (0.56–0.75)**	**0.67 (0.57–0.79)**	**0.70 (0.52–0.95)**	**0.62 (0.44–0.86)**
**Educational Level**				
Primary vs. None	**1.26 (1.07–1.47)**	**1.22 (1.02–1.45)**	**1.50 (1.09–2.07)**	**1.49 (1.06–2.11)**
Secondary vs. None	**1.49 (1.19–1.87)**	**1.36 (1.05–1.75)**	1.51 (0.94–2.44)	1.57 (0.94–2.62)
Tertiary vs. None	2.19 (1.44–3.32)	1.55 (0.98–2.44)	1.10 (0.53–2.26)	1.65 (0.71–3.79)
**Employment Status (employed vs. not)**	1.02 (0.90–1.17)	0.94 (0.81–1.09)	0.72 (0.54–0.98)	0.75 (0.53–1.05)
**Income (< = 5000 Ksh vs. >5001)**	**0.56 (0.35–0.89)**	0.66 (0.40–1.08)	1.27 (0.58–2.77)	**0.62 (0.44–0.86)**
**Attended ANC (No vs. Yes)**	**0.38 (0.34–0.44)**	**0.35 (0.31–0.41)**	**2.92 (2.05–4.15)**	**3.36 (2.29–4.93)**

### Testing uptake in HBCT

Generally, HBCT counseling (99.1%) and testing (96.2%) uptake was high (Table 1B). The difference in counseling and testing uptake between pregnant and non-pregnant women was small (Table 2). Pregnant women were more likely to agree to HIV testing in HBCT if they had at least primary education (vs. none) and had never attended ANC. They were less likely to agree to testing if they were older, married, and earning an income of ≤5000 Kenya shillings (~$58 USD) per month (Table 3).

### HIV prevalence

HBCT newly identified 3,653 (3.1%) women as HIV-positive with no significant difference between pregnant and non-pregnant women (p = 0.281) (Table 2). Combined with those who previously knew they were HIV-positive, HIV prevalence among these women of reproductive age was 6.9% and was the same in both pregnant and non-pregnant women. HIV prevalence among pregnant women was different according to whether they had attended ANC for the current pregnancy: 239/4394 (5.4%) among ANC attendees compared to 270/2993 (9.0%) among non-attendees.

As shown in Table 4, pregnant women were more likely to already know they were HIV-positive if they were older, married, and employed. Women with only primary education were less likely to have previously tested HIV-positive (adjusted odds ratio (AOR): 0.62, 95% confidence interval (CI) (0.44–0.87), as were those who had not yet attended ANC for this pregnancy (AOR: 0.61, 95% CI: 0.43–0.86). Pregnant women were much more likely to newly test positive in HBCT if they had not attended ANC during the current pregnancy (AOR: 6.85, 95% CI: 4.49–10.44) and less likely to newly test positive if they had ever previously tested for HIV (AOR: 0.49; 95% CI: 0.35–0.68).

**Table 4:** Factors associated with HIV prevalence among pregnant women.

Variables	Previously HIV positive		Newly HIV positive		Combined HIV prevalence (previous + newly)	
	UOR (CI)	AOR (CI)	UOR (CI)	AOR (CI)	UOR (CI)	AOR (CI)
**Age per 5 year increase**	**1.52 (1.38–1.67)**	**1.59 (1.44–1.76)**	0.98 (0.87–1.11)	1.00 (0.88–1.12)	**1.27 (1.18–1.37)**	**1.30 (1.21–1.41)**
**Marital Status**	**1.74 (1.29–2.36)**	**2.07 (1.48–2.88)**	1.04 (0.72–1.49)	0.94 (0.64–1.37)	**1.29 (1.02–1.63)**	**1.40 (1.08–1.81)**
**Educational Level**						
Primary vs. None	**0.59 (0.44–0.81)**	**0.62 (0.44–0.87)**	0.80 (0.55–1.14)	0.83 (0.57–1.21)	**0.70 (0.54–0.88)**	**0.76 (0.59–0.98)**
Secondary vs. None	0.31 (0.17–0.53)	0.30 (0.16–0.55)	0.65 (0.37–1.13)	0.72 (0.40–1.29)	**0.45 (0.30–0.67)**	**0.50 (0.33–0.76)**
Tertiary vs. None	0.13 (0.03–0.53)	0.10 (0.02–0.46)	0.13 (0.02–1.00)	0.17 (0.02–1.28)	**0.14 (0.05–0.46)**	**0.17 (0.05–0.55)**
**Employment Status (Employed vs. unemployed)**	**1.55 (1.15–2.10)**	**1.65 (1.16–2.35)**	0.92 (0.66–1.26)	0.86 (0.62–1.21)	1.23 (0.99–1.54)	1.17 (0.91–1.49)
**Income (< = 5000 Ksh vs. >5001)**	1.75 (0.64–4.81)	1.13 (0.37–3.43)	4.71 (0.65–34.02)	-	2.21 (0.90–5.45)	1.47 (0.57–3.82)
**Attended ANC (No vs. Yes)**	**0.76 (0.56–1.04)**	**0.61 (0.43–0.86)**	**8.31 (5.51–12.54)**	**6.85 (4.49–10.4)**	**1.71 (1.39–2.11)**	**1.53 (1.21–1.92)**
**Ever tested for HIV (No vs. Yes)**	*	*	**0.32 (0.23–0.43)**	**0.49 (0.35–0.68)**	*	*

NB: The above analysis excluded Webuye catchment area.

*Variable not included in the analysis

Overall, pregnant women in HBCT were more likely to be HIV-positive if they were older (AOR: 1.30, 95% CI: 1.21–1.41), married (AOR: 1.40, 95% CI: 1.08–1.81), and had not attended ANC for the current pregnancy (AOR: 1.53, 95% CI: 1.21–1.92). They were less likely to be HIV-positive if they had any education at all (Table 4).

## Discussion

The key finding of this paper is that pregnant women were more likely to test HIV-positive if they had not yet been to ANC for the current pregnancy. These data are important because they highlight a potentially very important gap in the PMTCT cascade which typically begins at the ANC; namely, women who have not been to ANC and are therefore less likely to get tested for HIV without a community-based intervention such as HBCT. Although HIV prevalence among pregnant women was not significantly different compared to non-pregnant women, our data suggest there is a high-risk and otherwise under-served subgroup of pregnant women seem not to be optimally accessing antenatal care.

Our study has important implications for improving HIV testing coverage among pregnant women in Kenya and other HIV endemic areas. Our data indicate that pregnant women in this population-based HBCT program had a high uptake of HIV testing, particularly among those who had never previously had an HIV test. Similar to HBCT studies in the general population, [13, 16] counseling and testing uptake among these pregnant women and women of reproductive age was high, and newly identified over 3,500 women, including about 250 pregnant women, as HIV-positive. These data highlight the need to intensify universal testing coverage of high-risk populations by going outside the health facility to reach these populations. These data suggest that ANC-based PMTCT programs are missing an important proportion of pregnant HIV-positive women who are therefore at risk of transmitting HIV to their babies and themselves going without antiretroviral treatment. If the goal of eliminating MTCT is to be realized, PMTCT programs may need to begin active case finding in the home and embrace a more community-oriented approach to service delivery.

Overall combined HIV prevalence in our study was slightly lower than the national ANC-based estimates among pregnant women (8.3%). This may be due to the inclusion of both high and low burden catchments in our population. Our findings also showed that increasing age is associated with a higher likelihood of pregnant women having been previously tested for HIV, and increased HIV prevalence. Previous studies in the general population have shown that HIV prevalence increases with age [17, 18] and efforts to continuously engage persons of all ages in HIV prevention are needed. We also noted that similar to other studies, [17] being married increased the likelihood of HIV infection. Low condom use and sexual concurrency (non-monogamy) among couples have been associated with these findings. [17] HIV prevention strategies that promote positive behavior change including improved communication among couples are necessary to mitigate the spread of HIV.

As expected, pregnant women with no education were more likely to be HIV positive. Previous findings have shown that individuals from a lower socio-economic strata have a higher HIV infection rate compared to those from higher socio-economic strata.[19] Education and economic empowerment of women is likely to reduce HIV infection rates including perinatal HIV, in these communities.[20, 21]

Optimal utilization of ANC services that have integrated PMTCT programs is essential for achieving the goal of zero MTCT HIV transmission.[6, 7, 13] Consistent with studies in this region our findings show that there are still large gaps in ANC coverage.[9, 22] The percentage of pregnant women who reported that they had not attended ANC was several times higher than the national estimates of 4.5%.[9] Our data are cross-sectional and ask about current pregnancy and ANC attendance while the Demographic Health Surveillance survey asks women about whether they had at least four ANC visits during their last live birth.[9, 23] Thus it is likely that a higher proportion of women in our study will have attended ANC by the time of their delivery compared to what we have documented here. Nevertheless, effective PMTCT requires early identification of pregnancy and early initiation of care and antiretroviral treatment. Our study provides indirect evidence that HBCT may be a good strategy to improve early pregnancy identification and initiation of care for pregnant women. Our study demonstrates similar findings as previous studies that have shown that income related inequalities greatly contribute to poor maternal healthcare seeking behaviors. Women of low literacy and income status are less likely to attend ANC than women of higher education level and income.[24–28] In addition, women from rural settings, report lower ANC attendance compared to those in urban settings.[22, 24–26, 28] Understanding the socio-economic and socio-cultural factors that create barriers to ANC coverage is important for defining appropriate health interventions.

This study has several strengths. We provide data on testing uptake among pregnant and non-pregnant women from six large catchments in HIV endemic areas covering a diverse cultural and ethnic population. These data are among the first to describe testing uptake and HIV prevalence amongst pregnant women in a population-based setting. Our study also has some limitations. Some of the variables including socio-demographic, socio-economic, and ANC attendance, were self-report measures, hence we cannot eliminate the possibility of reporting error/biases. Pregnancy was identified either by self-report or counselor identification of a gravid abdomen; the prevalence of pregnancy in this population may be therefore be subject to some misclassification bias. Pertinent data related to pregnancy such as parity and gestational age at the time of the HBCT encounter were not collected so we were not able to assess them as potential confounding factors. We acknowledge the drawbacks of secondary data analyses that limited the variables we included in the analysis. It is possible that ANC uptake and other service delivery has improved since the data were collected, and this may limit the generalizability of these findings to the present. Finally, the exclusion of Webuye catchment in the multivariable modeling because a number of key variables were not collected suggests that our findings should be interpreted with some caution.

In conclusion, our study highlights that PMTCT interventions initiated at the health facility may miss a substantial proportion of infections among pregnant women that could be identified in the home or community. This is potentially a serious missed opportunity for the prevention and elimination of perinatal HIV infection. Our findings show that pregnant women who had not attended ANC were less likely to have ever tested for HIV and several times more likely to newly test HIV-positive. In order to eliminate mother to child transmission there is need to identify more effective ways to promote early pregnancy identification, early ANC uptake, and early initiation of PMTCT regimens. In our study, HBCT uptake was high among pregnant women, representing an important opportunity for filling the gap in universal HIV testing coverage in this high-risk population. Our study shows that reaching into the home and community for case identification may improve PMTCT coverage and could contribute towards the goal of elimination of mother to child transmission.
